# The Association of Unreported Sleep Disturbances and Systemic Inflammation: Findings from the 2005-2008 NHANES

**DOI:** 10.1155/2018/5987064

**Published:** 2018-10-09

**Authors:** Jeff A. Dennis, Ahmad Alazzeh, Ann Marie Kumfer, Rebecca McDonald-Thomas, Alan N. Peiris

**Affiliations:** ^1^Department of Public Health, Texas Tech University Health Sciences Center, 3601 4th St., MS 9430, Lubbock, TX 79430, USA; ^2^Department of Pulmonary and Critical Care, East Tennessee State University, Quillen College of Medicine, VA Bldg. 1, Johnson City, TN 37614-0622, USA; ^3^Department of Internal Medicine, Texas Tech University Health Sciences Center, 3601 4th St., MS 9410, Lubbock, TX 79430, USA; ^4^Clinical Research Institute, Texas Tech University Health Sciences Center, 3601 4th St., MS 8183, Lubbock, TX 79430, USA

## Abstract

**Background/Objective:**

Sleep apnea is associated with elevated inflammatory markers. A subgroup of patients never report sleep disturbances to their physician. The inflammatory status of this subgroup is not known. The present study aims to evaluate two inflammatory markers, C-reactive protein (CRP) and red cell distribution width (RDW), in those with unreported sleep disturbances and compares these findings to those with and without reported sleep disorders. We also investigate the utility of RDW as an inflammatory marker in sleep disorders.

**Methods:**

Sample includes 9,901 noninstitutionalized, civilian, nonpregnant adults from the 2005-2008 National Health and Nutrition Examination Survey, a nationally representative, cross-sectional U.S. study. Sleep questionnaire and laboratory data were used to compare inflammatory markers (CRP and RDW) in five subgroups of individuals: reporting physician-diagnosed sleep apnea, reporting another physician-diagnosed sleep disorder, reported sleep disturbance to physician with no resulting diagnosis, unreported sleep disturbance (poor sleep quality not reported to physician), and no diagnosed sleep disorder or sleep disturbance.

**Results:**

Individuals with unreported sleep disturbance had significantly higher odds of elevated RDW (>13.6%) when compared to those without a sleep disturbance in adjusted models (OR=1.33). Those with unreported sleep disturbance had significantly higher odds of elevated CRP levels (>1 mg/L) than those without sleep disturbances (OR 1.34), although the association was not significant when adjusted for obesity and other controls.

**Conclusion:**

Self-identified unreported sleep disturbances are associated with significantly higher odds of elevated RDW than those without sleep disturbances. RDW may serve as a valuable indicator in identifying individuals at higher risk for sleep apnea and other sleep disorders.

## 1. Introduction

Obtaining optimal levels of sleep is associated with better health-related quality of life and reduced premature mortality risk [1]. Obstructive sleep apnea (OSA) is associated with hypertension, cardiovascular disease, pulmonary hypertension, neurocognitive effects, depressed quality of life, motor vehicle accidents, and disruption of the patients' bed-partners' sleep quality [2]. As many as 25% of adults are estimated to have OSA. It has been estimated that up to 80% of such adults do not have a formal diagnosis of sleep apnea [3]. The direct and indirect financial costs of failure to diagnose and treat sleep breathing disorders have been estimated at between 85 and 175 billion dollars [4].

Untreated sleep breathing disorders and obstructive sleep apnea are associated with an increase in inflammatory markers [5]. A variety of inflammatory markers such as C-reactive protein (CRP), tumor necrosis factor alpha, and interleukin-6 have been used to estimate inflammation in untreated sleep apnea [5]. Increased levels of CRP are recognized as an independent risk factor for future cardiac events both in healthy individuals and in patients with cardiovascular disease [5–7]. Moreover, elevated CRP has also been linked to increased risk of type 2 diabetes [8]. While CRP has been a more established marker for inflammation, red cell distribution width (RDW) has emerged as a marker independently associated with increased inflammation [9]. In some cases RDW has been superior to CRP in predicting cardiovascular mortality [10]. In patients with OSA, the majority of studies have shown the RDW value to correlate with established indices that reflect the severity of OSA and risk of cardiovascular disease [11–13]. In patients with OSA, the potential benefit of continuous positive airway pressure (CPAP) in reducing inflammatory markers has been noted [14, 15]. However, sleep disorders other than OSA may also carry increased risk of adverse health outcomes, although few, if any, studies have examined inflammatory markers in sleep disorders outside of OSA [16].

Many patients who experience sleep disturbances never report them to their physicians [17–20]. Estimates suggest as much as 5% of the U.S. adult population may have undiagnosed sleep apnea that warrants medical treatment [21]. There is a paucity of data studying the inflammatory status in these individuals. We hypothesized that inflammatory markers will be elevated in such sleep disturbances and this constitutes a high-risk group. The present study was undertaken to compare and contrast CRP and RDW responses in individuals with unreported sleep disturbances to those diagnosed with sleep disorders and those reporting no sleep problems. We hypothesized that individuals with unreported sleep disturbances have increased inflammatory markers compared to those not reporting sleep problems.

## 2. Materials and Methods

This study uses cross-sectional data from the National Health and Nutrition Examination Survey (NHANES) 2005-2008, which contained a detailed sleep questionnaire in addition to the standard demographic, laboratory, and examination data collected each two-year cycle. A final sample of 9,901 respondents was used, representing all cases with complete information on all independent and dependent variables. A majority of those excluded from the final sample were 1,216 individuals (10% of original data) with no recorded value for CRP, RDW, or both dependent variables. NHANES uses random subsamples for some laboratory testing to reduce burden on participants, which may account for some of these 1,216 cases [22]. These cases are further discussed in the Results section below. Additionally, 384 women (3% of sample) who reported they were pregnant were excluded since sleep patterns and biomarkers may be atypical in pregnancy. Other exclusions (<1% of the sample) included individuals without reported data for BMI, sleep quality, or hours of sleep.

NHANES data is deidentified and publicly available, and all analyses for this study report on aggregate findings. As a result, the first author's Institutional Review Board declined to review the project due to not meeting the criteria for human subject research.

Diagnosed sleep disorders were self-reported in the sleep questionnaire via the question “have you ever been told by a doctor or other health care professional that you have a sleep disorder?” [23], a follow-up question asked for the specific diagnosis. For analytic purposes, sleep disorders were divided into two categories, those reporting a diagnosis of sleep apnea and those reporting any other diagnosed disorder, and comprised those reporting a diagnosis of insomnia, restless legs, or an “other,” nonspecified, sleep disorder. The “reported sleep disturbance” group consisted of individuals who responded that they had reported sleep problems to their doctor but were not given a sleep disorder diagnosis.

Remaining individuals who had not reported sleep disturbance to a physician were categorized based on self-reported sleep quality. Using the method from Liu and colleagues [24], individuals who reported that they “almost always” (defined as 16-30 times per month) had at least one of a list of 8 sleep quality issues were categorized as having poor sleep quality. Items from this inventory include (1) trouble falling asleep, (2) waking up during the night, (3) waking too early, (4) feeling unrested, (5) feeling overly sleepy, (6) feel didn't get enough sleep, (7) have leg jerks while sleeping, and (8) have leg cramps while sleeping [23]. Individuals who fit this definition of having poor sleep quality, but who said they had never reported sleep problems to their doctor, are categorized as having “unreported sleep disturbance.” The “no sleep problem” group was defined as those individuals who had never reported sleep problems to their doctor and did not report poor sleep quality.

The above criteria produced 5 comparison groups: (1)* reported sleep apnea diagnosis* (self-reported physician diagnosis), (2)* reported sleep disorder diagnosis other than apnea* (self-reported physician diagnosis), (3)* reported sleep disturbance* (reported to physician with no resulting diagnosis), (4)* unreported sleep disturbance* (poor sleep quality not reported to physician), and (5)* no reported sleep problems or diagnoses*.

CRP was coded as a dichotomous variable representing “average to high risk” when CRP > 1 mg/dL, a value past studies using NHANES have defined as “clinically elevated” [25]. CRP >3 mg/dL, a common high risk cutoff, was not prevalent enough for meaningful analysis (about 1.3% of the population). RDW was also coded as a dichotomous variable using a cutoff of RDW ≥13.6%, a value that has been associated with increased cardiovascular disease risk in sleep apnea patients [12]. Obesity was defined using the standard cutoff of body mass index of 30 kg/m^2^ or higher. Hours of sleep were self-reported in NHANES and were coded into three groups: less than 7 hours, 7-8 hours, and 9 or more hours.

The breadth of NHANES also allows for inclusion of other comorbidities as control variables. A comorbidity index was constructed as a count of self-reported physician diagnosis of a variety of factors found to be associated with sleep apnea and inflammation. This variable ranges from 0 to 7, and counts reports of physician diagnoses of diabetes, asthma, hypertension, high cholesterol, coronary heart disease, congestive heart failure, and depression, as measured by a score of 10 or more on the PHQ-9 Questionnaire [26–28]. We also use available variables reporting current use of cholesterol or hypertension medication, given the association of medications for these conditions in reducing CRP [29]. However, use of blood pressure medication was not a significant predictor of elevated CRP or RDW and was dropped from the final adjusted models.

Logistic regression odds ratios and 95% confidence intervals compared differences in odds of elevated CRP & RDW between sleep groups, controlling for race/ethnicity, gender, age, obesity, comorbidity index, current use of cholesterol medication, and hours of sleep. Statistical significance was set at p<0.05. Statistical analyses used Stata 14.0 with population weighting to account for the complex survey design of NHANES [23].

## 3. Results

Table 1 shows weighted sample characteristics for the analysis sample. The nationally representative nature of NHANES allows for estimation of the size of these subgroups in the U.S. adult population at the time of survey, that is, from 2005-2008. Over 7% (estimated 16.2 million adults) of the sample had some type of diagnosed sleep disorder, with slightly more than 60% of those diagnosed with sleep apnea. Approximately 11% (estimated 24.7 million adults) of the sample had sleep disturbances not reported to a physician and 18% (estimated 40.3 million adults) had reported sleep disturbance to their physician.

Additional analysis (See the Appendix) compares the analysis sample to the 1,216 individuals dropped because they did not have CRP and RDW measures. The 1,216 individuals without CRP or RDW values were younger, more likely to be African American, less likely to have a sleep apnea diagnosis, and less likely to get 7-8 hours sleep per night than those included in the analysis sample. The two groups did not differ on gender or obesity status. Despite some demographic differences, and given that NHANES randomly selects subsamples for some laboratory tests, no evidence suggests that the absence of this group would markedly change any of the relationships between sleep and inflammation.

Table 2 presents percentages of each sleep group for values above the defined thresholds for CRP and RDW and for obesity. Elevated CRP occurred at a significantly higher rate in respondents with sleep apnea, other sleep disorder, and reported sleep disturbance, compared to no sleep problems. Respondents with unreported sleep disturbance did not differ significantly on CRP at the bivariate level from those with no sleep problems. Elevated RDW levels were also highest among those with diagnosed sleep disorders, followed by those with reported and unreported sleep disturbance, both of whom had rates of elevated RDW significantly higher than those with no sleep problems. Those with no sleep problems had significantly lower prevalence of elevated RDW compared to all other groups. Those with sleep apnea were twice as likely to be obese compared to those with reported and unreported sleep disturbance and those with no sleep problems. No significant differences existed in obesity rates among the two sleep disturbance groups and the respondents with no sleep problems.

Table 3 presents logistic regression odds ratios predicting CRP > 1 mg/dL. The unreported sleep disturbance group had increased odds of elevated CRP (OR=1.34, 95% CI: 1.02, 1.76) compared to those with no sleep problems, although this association was not significant after adjusting for obesity, comorbidities, cholesterol medication, and hours of sleep. Only individuals with reported sleep disturbance had significantly higher odds (OR=1.42, 95% CI:1.17,1.72) of elevated CRP compared to those with no sleep problems, after adjusting for controls in Model 2.

Table 4 shows logistic regression odds ratios predicting RDW≥13.6%. The unreported sleep disturbance group exhibited higher odds of elevated RDW (OR=1.33, 95% CI: 1.04, 1.70) compared to those with no sleep problems after adjusting for obesity and hours of sleep. Individuals with sleep apnea similarly exhibited higher odds (OR=1.37, 95% CI: 1.02, 1.85) of elevated RDW≥13.6% compared to those with no sleep problems.

## 4. Discussion

Our study was unique in that we examined inflammatory markers in patients with sleep disturbance (reported and unreported) and compared them to patients reporting diagnosed sleep disorders and those with no reported sleep problems or diagnoses. To our knowledge, this is the first study to demonstrate that individuals with unreported sleep disturbances may have increased odds of elevated RDW. Our data supports the hypothesis that the unreported sleep disturbance group have higher inflammatory markers than the group without sleep problems. Given that RDW is collected as part of a routine complete blood count (CBC), it may have utility in screening for sleep disorders.

Sleep disorders promote a low-grade inflammatory status by increasing circulating proinflammatory cytokines (IL-1*β*, IL-6, IL-17A, TNF-*α*) and CRP [5, 6, 30]. Sleep-deprived people were found to have elevated numbers of monocytes, neutrophils and phagocytic cells in peripheral circulation that are responsible for the production of IL-6 and TNF-alpha messenger RNA. Proinflammatory cytokines present a peak in early nocturnal sleep in correlation with the accumulation of molecules such as adenosine or reactive oxygen species [31]. Shift workers exposed to irregular sleep schedules resulting in sleep deprivation and misalignment of circadian rhythms, have increased CRP levels and insulin resistance [32]. It has also been argued that sheer stresses, or physical stress forces, associated with increased blood pressure in sleep-deprived people may activate inflammatory mediators [31].

CRP is a well-characterized inflammatory marker associated with cardiovascular risk in those with OSA [33, 34]. However, hs-CRP is independently associated with obesity, and some studies have found that the increased CRP levels in those with OSA are explained by obesity [35, 36]. Our study found that when obesity and other controls were added as covariates, the odds of having a CRP level >1 mg/dL in the sleep apnea group decreased substantially, to nonstatistical significance (OR=2.46 compared to OR=1.33).

RDW is a measure of the heterogeneity of red blood cells that may have greater future utility as an inflammatory marker. It is possible that an increased RDW value is an index of ineffective erythropoiesis [37]. A high RDW has been linked to an increasingly diverse series of adverse outcomes spanning across many disease states [38]. Studies on humans and animals showed clear evidence of increased RDW in responses to low oxygen tension [39]. Several studies have found RDW elevation to be associated with the severity of OSA and the risk of developing cardiovascular disease [11, 12]. Studies clearly demonstrated an association between RDW values and sleep apnea syndrome severity. Moreover, RDW values changed significantly after positive airway pressure treatment [13]. However, relatively few studies compare RDW levels in individuals with sleep disturbances exclusive of obstructive sleep apnea. As such, our finding demonstrating that RDW values are significantly elevated in those with sleep disorders and sleep disturbances is interesting. We believe that RDW is a potentially significant laboratory metric that can provide diagnostic synergy when added to screening for sleep disorders.

### 4.1. Obesity and Sleep

The association of obesity with OSA is well characterized. While it is estimated that 70% of patients with OSA are obese, 30% of patients with OSA lack this well-known risk factor and may experience delayed evaluation [40]. Our analysis found that even after controlling for obesity, those with sleep disorders and reported sleep disturbances showed increased RDW and CRP levels compared to those without sleep disturbances. Those with unreported sleep disturbances had less than half the prevalence of obesity when compared to those with a diagnosed sleep disorder (Table 2 - 32.22% obese compared to 68.93% obese, respectively). This suggests that although obesity is among the key risk factors for sleep apnea, clinicians should not consider obesity a necessary factor for proactive sleep disorder screening.

A strength of this study is that we included several groups that have not received much attention in terms of sleep disorders, including patients not reporting their sleep symptoms to clinicians. Only 4% of referrals to a sleep center occurred because the clinician obtained information of sleep disturbance [41]. Qualitative evidence suggests discord in doctor-patient interactions regarding sleep problems [42], such that identification of new clinical markers may be beneficial. It remains to be seen if earlier identification, attention, and intervention in patients with unreported sleep symptoms will have health benefits.

### 4.2. Limitations

The NHANES sleep questionnaire did not ask about use of sleep disorder treatments such as CPAP, nor does it have data on severity of sleep apnea in the sleep disorder group. Therefore, this study could not account for the severity of the sleep apnea or effect of treatment of sleep apnea. Given the cross-sectional and deidentified nature of the study data, we could not follow up on patients with sleep disturbances to assess patient outcomes. However, despite these limitations, we believe the nationally representative sample provides some insight into potential associations between unreported sleep disturbance and elevated RDW.

## 5. Conclusions

Compared to those without significant sleep disturbances, both reported and unreported sleep disturbances groups had increased RDW. Since RDW is readily obtainable in a routine CBC, it may identify a population at risk for sleep apnea and merits further study. It also raises the possibility that treatments for sleep disturbances with such agents as Ramelteon may have dual benefits on inflammation as well as sleep [43].

## Figures and Tables

**Table 1:** Analysis sample frequencies and weighted percentages.

Variable	Unweighted Frequency	Weighted Percent
Gender		

Female	4,894	50.96

Male	5,007	49.04

Race/Ethnicity		

NH white	4,697	71.22

NH black	2,159	10.85

Mexican	1,901	8.19

Other Hispanic	755	4.24

Other race	389	5.50

Age		

18-44	4,526	48.23

45-64	3,084	35.45

65 plus	2,291	16.32

Sleep categories		

Sleep apnea	411	4.56

Other sleep disorder	293	2.80

Reported sleep disturbance	1,596	18.30

Unreported sleep disturbance	1,167	11.19

No sleep problems	6,434	63.15

Obesity		

Not obese	6,454	66.73

Obese	3,447	33.27

Average Hours of sleep		

Less than 7	3,831	36.72

7-8 hours	5,303	56.45

9 or more hours	767	6.83

Comorbidity scale - Mean (SD)	9,901	0.86 (0.02)

Taking prescription for hypertension		

No	7,027	77.79

Yes	2,415	22.21

Taking prescription for high cholesterol		

No	8,349	85.75

Yes	1,552	14.25

Data source: NHANES 2005-2008.

N=9,901.

**Table 2:** Weighted percentages with CRP>1 mg/dL, RDW*⩾*13.6%, and obesity, by sleep category.

	CRP>1 mg/dL	Sig	RDW≥13.6%	Sig	Obese	Sig
	Pct. (SE)		Pct. (SE)		Pct. (SE)	
Sleep apnea	15.88 (2.06)	*∗*	19.03 (2.17)	*∗*	68.93 (3.43)	*∗*

Other sleep disorder	15.79 (2.67)	*∗*	18.72 (2.55)	*∗*	44.02 (3.21)	*∗*

Reported sleep disturbance	12.42 (0.89)	*∗*	15.40 (1.03)	*∗*	33.25 (1.49)	

Unreported sleep disturbance	9.7 (1.06)		15.34 (1.26)	*∗*	32.22 (2.06)	

No sleep problems	7.5 (0.33)		11.70 (0.65)		30.41 (1.24)	

Full sample	9.26 (0.33)		13.32 (0.53)		33.27 (0.94)	

N=9,901; *∗*significantly different from No sleep problems at p<0.05 (Wald test).

Data: NHANES 2005-2008.

**Table 3:** Logistic regression predicting odds of CRP>1 mg/dL.

	Model 1 OR^a^	95% CI	p value	Model 2 OR^b^	95% CI	p value
Sleep apnea	2.46	[1.72,3.50]	<0.001	1.33	[0.90,1.95]	0.145

Other sleep disorder	2.03	[1.30,3.17]	0.003	1.52	[0.98,2.35]	0.061

Reported sleep disturbance	1.56	[1.30,1.87]	<0.001	1.42	[1.17,1.72]	0.001

Unreported sleep disturbance	1.34	[1.02,1.76]	0.034	1.23	[0.94,1.62]	0.122

N=9,901.

Data source: NHANES 2005-08.

Reference group: no sleep disorder or disturbance.

^a^adjusted for gender, race/ethnicity, and age.

^b^adjusted for gender, race/ethnicity, age, obesity, hours of sleep per night, comorbidity index, and taking cholesterol medication.

**Table 4:** Logistic regression predicting odds of RDW*⩾*13.6%.

	Model 1 OR^a^	95% CI	p value	Model 2 OR^b^	95% CI	p value
Sleep apnea	1.91	[1.38,2.65]	<0.001	1.37	[1.02,1.85]	0.035

Other sleep disorder	1.60	[1.16,2.20]	0.006	1.33	[0.94,1.89]	0.108

Reported sleep disturbance	1.26	[1.01,1.58]	0.038	1.15	[0.93,1.44]	0.191

Unreported sleep disturbance	1.41	[1.09,1.82]	0.01	1.33	[1.04,1.70]	0.025

N=9,901.

Data source: NHANES 2005-08.

Reference group: no sleep disorder or disturbance.

^a^adjusted for gender, race/ethnicity, and age.

^b^adjusted for gender, race/ethnicity, age, obesity, hours of sleep per night, comorbidity index, and taking cholesterol medication.

**Table 5:** Frequencies and weighted percentage comparison between analysis sample and missing cases.

	Analysis Sample		Missing Cases			
Variable	Unweighted Frequency	Weighted percent	Unweighted Frequency	Weighted percent	Chi square	p value
N	9,901		1,216			

Gender						

Female	4,894	50.96	629	51.40	0.05	0.856

Male	5,007	49.04	587	48.60		

Race/Ethnicity						

NH white	4,697	71.22	490	61.09	76.33	<0.001

NH black	2,159	10.85	361	21.75		

Mexican	1,901	8.19	212	7.02		

Other Hispanic	755	4.24	95	4.11		

Other race	389	5.50	58	6.03		

Age						

18-44	4,526	48.23	628	62.60	58.43	<0.001

45-64	3,084	35.45	266	22.49		

65 plus	2,291	16.32	322	14.92		

Sleep categories						

Sleep apnea	411	4.56	37	2.37	14.55	0.019

Other sleep disorder	293	2.80	47	3.81		

Reported sleep disturbance	1,596	18.30	174	15.45		

Unreported sleep disturbance	1,167	11.19	133	10.51		

No sleep problems	6,434	63.15	825	67.87		

Obesity						

Not obese	6,454	66.73	467	66.52	0.01	0.916

Obese	3,447	33.27	245	33.48		

Average Hours of sleep						

Less than 7	3,831	36.72	478	39.04	17.24	0.005

7-8 hours	5,303	56.45	612	50.45		

9 or more hours	767	6.83	124	10.51		

Comorbidity scale - Mean (SD)	9,901	0.86 (0.02)	1,216	0.69 (0.05)	11.38	0.002

Taking prescription for hypertension					7.15	0.029

No	7,027	77.79	879	82.26		

Yes	2,415	22.21	294	17.74		

Taking prescription for high cholesterol					9.23	0.005

No	8,349	85.75	1044	89.93		

Yes	1,552	14.25	172	10.07		

Data source: NHANES 2005-2008.

## Data Availability

NHANES data used in this manuscript is freely available via the Centers for Disease Control website (https://www.cdc.gov/nchs/nhanes/index.htm).

## Appendix

See Table 5.
